# Strategies to avoid blacklisting: The case of statistics on money laundering

**DOI:** 10.1371/journal.pone.0218532

**Published:** 2019-06-26

**Authors:** Joras Ferwerda, Ioana Sorina Deleanu, Brigitte Unger

**Affiliations:** 1 Utrecht University School of Economics, Utrecht University, Utrecht, The Netherlands; 2 Utrecht University School of Governance, Utrecht University, Utrecht, The Netherlands; University of Texas at San Antonio, UNITED STATES

## Abstract

Financial and legal entities (e.g. banks, casinos, notaries etc.) have to report money laundering suspicions. Countries’ engagement in fighting money laundering is evaluated–among others–with statistics on how often these suspicions are reported. Lack of compliance can result in economically harmful blacklisting. Nevertheless, these blacklists repeatedly become empty–in what is known as the emptying blacklist paradox. We develop a principal-agent model with intermediate agents and show that non-harmonized statistics can lead to strategic reporting to avoid blacklisting, and explain the emptying blacklist paradox. We recommend the harmonization of the standards to report suspicion of money laundering.

## Introduction

The Financial Action Task Force (FATF) was founded by the G-7 in 1989 and was mandated to set international standards and coordinate the global effort to combat money laundering. To this end the FATF evaluates countries’ anti-money laundering (AML) policies and their effectiveness [1]. Countries that receive a negative evaluation from the FATF face the risk of being blacklisted. Blacklisting carries a large reputational cost and may even lead to an economic embargo [2,3]. Nevertheless, the success of this strategy is heavily contested [2,4]. Soon after the FATF published a new blacklist, countries removed themselves from it by providing the FATF with evidence that they are meeting the necessary standards. Paradoxically, a world with no blacklisted countries is still a world rifle with large scale money laundering schemes, as field experiments [5] and whistleblowers (e.g. Panama Papers, WikiLeaks, Luxembourg Papers) show.

This paper adheres to the growing body of literature that questions the effectiveness of the global AML strategy [5–12] and to the literature questioning the reliability of national statistics caused by international standards and evaluations [13–16]. By means of a principal-agent model with multiple agents that multi-task [17], we show that the absence of a harmonized definition for a suspicion report (SR)–one of the main indicators used by the FATF as metric of the engagement of the private sector in exposing money laundering–may explain the blacklisting paradox. In the absence of a harmonized legal definition of an SR, countries can avoid negative evaluations without burdening the private sector with the real cost of detecting money laundering. The distinguishing mark of our model is that the welfare of the intermediate agent (the state) depends on the welfare of the other agents (the reporting entities), as the state is not only the enforcer of the FATF regulations, but also the enforcer of the wishes of its constituents.

There are at least six aspects of the definition of an SR that are vulnerable to abuse for statistical purposes: (1) the type of SR (e.g. cash, wire, checks); (2) the subjective grounds of suspicion (the level of necessary knowledge when defining a transaction as suspicious); (3) the objective grounds of suspicion (the reporting threshold of the amount of money involved in a transaction); (4) the definition of a transaction (the activities which constitute a transaction affect the applicability of reporting); (5) the inclusion of attempt (including the attempt of a transaction affects the number of reports); and (6) the data collection methodology (using separate versus bundled SRs) [18]. Even in the absence of *mala fide* intent, these six vulnerabilities carry on in national statistics making it difficult for the FATF to evaluate countries on the basis of their true effort to combat money laundering, and consequently enlarges the gap between the metric of effort ‘on paper’ and ‘in practice”.

The paper consists of six sections. The following section introduces the literature that has theoretically discussed the strengths and weaknesses of the FATF blacklists strategy. Section 3 exposes the blacklist paradox and the incomparable nature of one of the parameters used by the FATF to rank countries on their efforts to prevent money laundering. It also introduces a repurposed three-layered principal-agent model where the principal is the FATF, the intermediary agent is the national government (which includes the policymaker, the supervisor and the Financial Intelligence Unit (FIU) mentioned by [6,7]), and the final agent is an obliged entity. This modeling choice allows the analysis of the dynamics between the FATF and the countries subjected to its mandate through the standard lens of a principal-agent model (also employed by [6,7,10,19]), offers a simple explanation for why the FATF blacklists empty and helps identify the potential weaknesses, as well as, provides the strategies for remedy. Section 4 details the consequences derived from the model, namely that, as long as strategic behavior through non-harmonized statistics is cost efficient, using these statistics for the purpose of blacklisting will not prevent money laundering and will further reduce the informational quality of national statistics on money laundering. Section 5 puts forward improvements to the policy of the FATF: (1) the harmonization of the definition of an SR and (2) the provision of direct metrics of engagement for the private sector. Finally, section 6 concludes.

## Literature overview

Doubts on the effectiveness of the FATF strategy were voiced early on in the academic community. [6] suggested the paradox is a result of the “zero tolerance policy”. Obliged entities (i.e. organizations legally designated to monitor their customers and report suspicion of money laundering taking place through their dealings) have to make a trade-off: they can either report less (thereby saving on the associated detection and reporting costs and on the costs of losing high net worth individuals as clients), and accept the possibility of being sanctioned; or they can report more and accept the extra costs. Because law enforcement cannot distinguish between true reports and false positives received from the obliged entities, Takats showed that the amount of reporting went up as soon as the sanctions were raised but that this did not necessarily lead to more convictions of money launderers. Essentially, when sanctions are too high, law enforcement is flooded with low quality reports [20].

[7] proposed a three-player principal agent model to analyze the incentive problems present in the AML system. In their model, the principal–the policy maker–wants to maximize the true suspicious reports while being sensitive to the costs incurred by the financial institutions. The policy-maker cannot observe the true effort exercised by the agent–the obliged entities. Consequently, he hires an intermediary agent–a supervisor–whose job is to reduce the information asymmetry. Assuming that the supervisor is not a typical shirking agent, [6] concluded that supervision is more effective than fines.

What [6,7] miss, however, is a good representation of the locus of compliance. Factually, the real locus of compliance is at the business-actor level (cf.[5]), but in the grand strategy of the FATF, the locus of compliance rests at the state-actor level. Sanctions imposed on state actors impact thus the reporting of the private sector, though not according to the analysis of [6], and absent international pressure, countries may not aim to maximize the number of true suspicion reports altogether, contrary to the assumptions of [7]. Placing the state-agent in the role of an agent rather than in the role of the principal, however, requires the willingness to question the real motives of a government. Though recent studies [13,14,16] support the need to question the objectivity of national statistics when governments face diverging national and international pressures, this possibility has not settled yet in the mainstream literature.

## Materials and methods

### The paradox of money laundering with empty blacklists

The success of the global AML strategy heavily depends on the adequate participation of all countries. To this end, the FATF is mandated to set the standards for participation, monitor, and assess the efforts undertaken by countries in combating money laundering [21,22]. Since these efforts are not directly observable or directly enforceable, the FATF performs regular checks to measure effort and compiles its findings in a series of ‘Mutual Evaluation’ and ‘Follow-Up’ reports. The FATF incentivizes countries to cooperate by means of a naming-and-shaming strategy [23]. Its ultimate “big gun” is the blacklisting of countries that are found to be non-compliant in the course of a round of mutual evaluations–with serious negative consequences for the country’s reputation, trade and business relations. When Pacific Island Nauru was blacklisted for money laundering, US authorities cautioned its banks and asked them to take special precautions when dealing with Nauru. Eventually, several banks and financial institutions refused to transact with Nauru. Economic isolation is particularly harmful for small economies [24].

**Fig 1:**
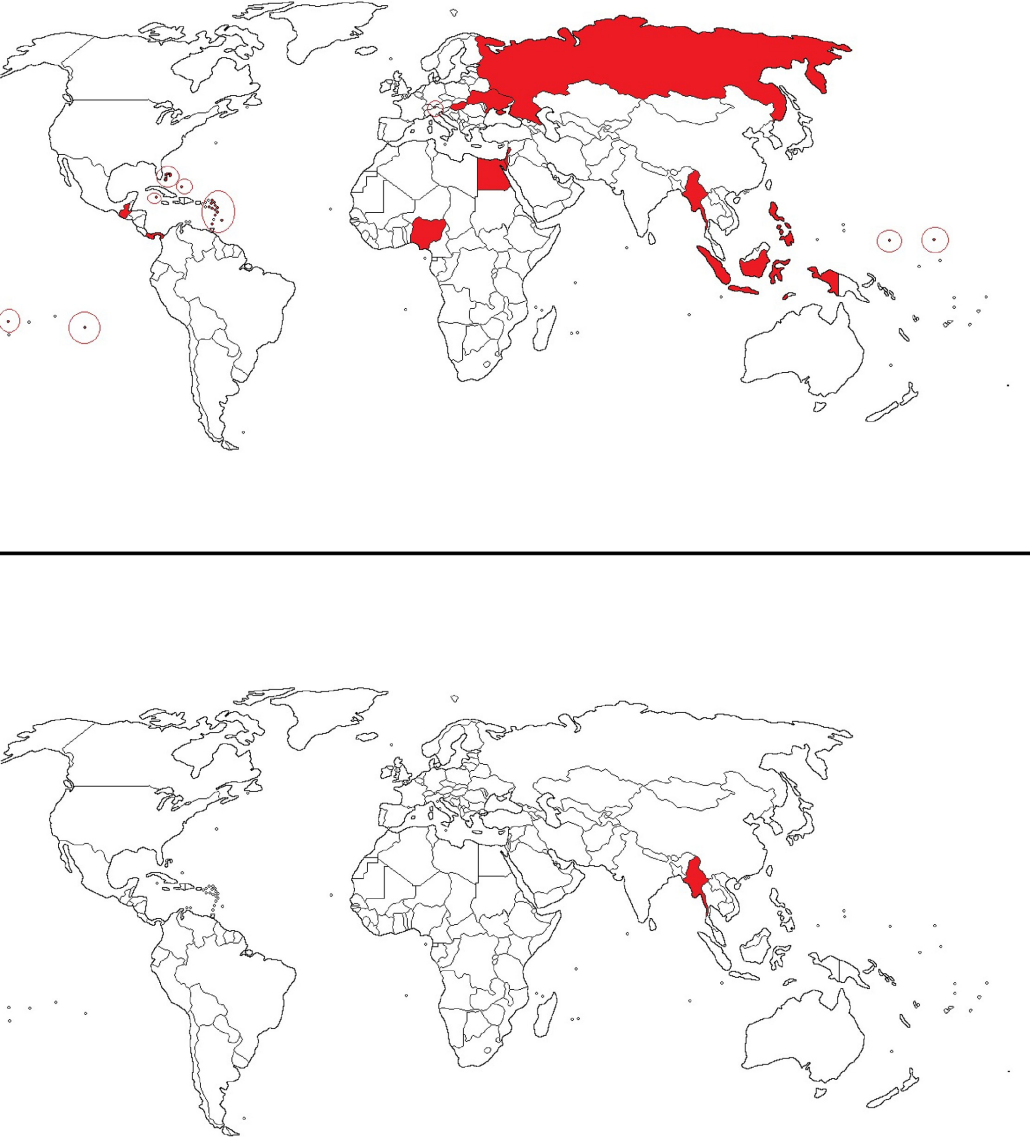
Blacklisted countries: 2000 and 2001 in the upper chart, 2006 in the lower chart. **Blank map political world territories, shared under CC-BY-SA 4.0 license, altered for illustrative purposes.** The blacklisted countries in 2000 and/or 2001 included: Egypt, Nigeria, Cook Islands, Indonesia, Myanmar, Marshalls lands, Nauru, Niue, Philippines, Hungary, Liechtenstein, Russia, Ukraine, Israel, Lebanon, Bahamas, Cayman Islands, Dominica, Grenada, Guatemala, Saint Kitts and Nevis, Panama and Saint Vincent and the Grenadines. Blacklisted in 2006: Myanmar.

The FATF published its first blacklist (officially known as the list of Non-Cooperative Countries and Territories) in 2000 with a total of 15 countries, mainly small islands in the Caribbean and the Pacific [25]. The 2001 blacklist contained 19 countries. Their numbers declined steadily until Myanmar was removed in October 2006 (Fig 1).

Following criticism on its blacklisting strategy [2], the FATF published in 2009 a revised blacklist, which initially distinguished four groups of non-compliant countries, ranging from highly non-compliant to little non-compliant. In 2013, the FATF has published a simplified version where it recognized only two groups of non-compliant/non-cooperative jurisdictions [26]. The FATF soon saw the new list empty. The 2011 FATF list consisted of only 10 countries, down from 28, in 2010 as Angola, Antigua and Barbuda, Azerbaijan, Ecuador, Greece, Indonesia, Morocco, Nepal, Nigeria, Pakistan, Paraguay, Qatar, Sao Tomé and Principe, Sudan, Thailand, Trinidad and Tobago, Turkmenistan, Ukraine and Yemen were removed while Cuba was placed on the list. The 2015 FATF list mentioned only 5 countries: Iran, North Korea, Algeria, Ecuador and Myanmar [27] while in 2018 the FATF only mentions North Korea and Iran.

At the same time however, we have seen the largest leaks on off-shores, shell companies and money laundering schemes (e.g. Swiss Leaks, Luxembourg Leaks, Panama papers), therewith supporting the critics of the efficiency of the current black/grey lists of the FATF [28]. Additionally, using randomized controlled trials, [5] showed that FATF standards are often not enforced in the case of anonymous shell companies. Moreover, countries where compliance to the FATF standards was lowest in practice were not black-listed and some had never even been on a black or a grey list–e.g. Danske Bank in Denmark [29].

### Comparing non-harmonized statistics

SRs represent one of the parameters used by the FATF as metric for the responsiveness of the private sector to the money laundering threat. There are four different types of SR. The most common is the Suspicious Transaction Report (STR) which is a report obliged entities send to the FIU at the sight of a transaction they suspect is done to launder money. The Suspicious Activity Report (SAR) allows also non-transactions to be reported–e.g. opening a bank account. The Unusual Transaction Report (UTR) broadens the definition of suspicion–e.g. a transaction where there is no information or red flag suggesting money laundering but that is unusual given a client’s transacting patterns. Finally, the Cash Transaction Report (CTR) reports only transactions in cash. [18] show that, in practice, these reports do not respect their limitations and that some countries use multiple types of reports, as shown in Table 1. They conclude that comparing countries on the basis of SRs would be imprudent.

**Table 1:** SRs in 7 European countries in 2015, in absolute terms and per capita.

Country	No. STRs	/1000ppl	No. UTRs	/1000ppl	No. SARs	/1000ppl	No. CTRs	/1000ppl	Source
**Denmark**	15,619	2.77							[30]
**Germany**	29,108	0.36							[31]
**Hungary**	8,369	0.85							[32]
**Latvia**	23,061	11.59	9,904	4.98					[33]
**Poland**	40,331	1.04			2,864	0.07	28,900,000	748.32	[34]
**Switzerland**					2,367	0.29			[35]
**UK**					381,882	5.94			[36]

Table 1 reveals the SR statistics in 2015 for a sample of European countries: Denmark, Germany, Hungary, Latvia, Poland, Switzerland and the United Kingdom. This sample was constructed to illustrate the diversity of SRs and that in spite of the differences SRs are used by the FATF to compare countries in their effectiveness and compliance.

In 2006, the FATF compared the Danish level of reporting to that of Hungary and Switzerland while acknowledging that it is not a good basis for comparison. In Denmark filing an SRT requires suspicion of money laundering in connection with a criminal offence punishable by imprisonment of one year or more, [37] while in Hungary “any data, facts or circumstances indicating money laundering” [38] are sufficient to file an STR. Similarly, in 2010 the FATF expressed concerns about the effectiveness of the German reporting system after comparing the number of STRs with those in the United Kingdom [39]. The FATF agreed that differences may be due to the methods used to count SRs (e.g. different levels of suspicion required, comparing SARs to STRs, different scopes of application for the reporting obligations), but nevertheless concluded that Germany had too few STRs [39]. Interestingly, had the German STRs been compared to those of Switzerland and Denmark, the FATF could have reached the exact opposite conclusion.

### Modeling the strategic response to blacklisting

The literature [6,7] has described the FATF’s blacklisting strategy using the contractual lens of the principal agent theory. The standard principal-agent model assumes the principal delegates a task to the agent [40–42]. The successful completion of the task increases the payoff of the principal but does not directly increase the payoff of the agent as the agent needs to exert costly effort to successfully complete the task. In order for a lucrative relationship to exist between the principal and the agent, the principal must create the incentives for the agent to not slack. The relationship becomes problematic with asymmetric information and conflicting interests [43]–i.e. when the agendas of the agent and principal are not aligned, and when the principal cannot correctly assess whether the agent is slacking. We repurpose the three-layered principal-agent model such that the principal is the FATF, the intermediary agent is the national government, and the final agent is the obliged entity.

**Fig 2:**
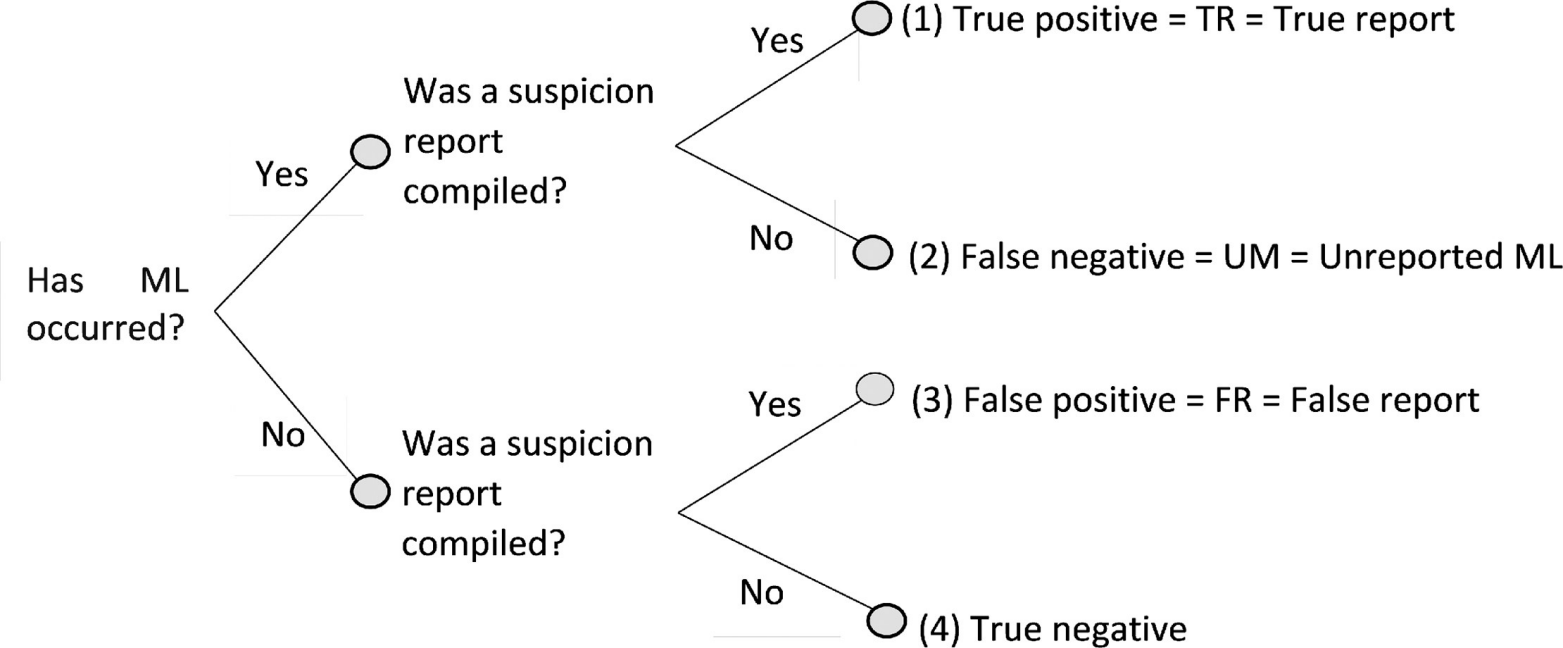
Event tree of reporting money laundering. ML–money laundering.

Fig 2 introduces the event tree on which our model is based. Asymmetric information assumes that the reporting entities have good indicators to answer the first question, while the principal can infer the answer only by observing the number of total suspicion reports (true reports (*TR*) and false reports(*TR*)). This inference is subject to error.

Fig 2 shows that the total Number of Suspicion Reports is

SR=TR+FR (1)

Fig 2 also shows that the total number of Money Laundering attempts is

MA=TR+UM (2)

Let us assume, for simplicity, that the payoff function of the principal only depends on the number of true SRs registered in each country. As shown earlier, in practice, the FATF downgrades countries on the basis of the basis of the number of SRs put forward by their obliged entities. The payoff function of the principal is

$$u(i)=max({TR}_{i})$$ (3)

where *i* ∈ {1,*n*} is the number of countries, and *TR* is the number of suspicionreports correctly reporting a money laundering transaction or activity.

In order to effectively combat money laundering, countries must exert a certain degree of effort. Reporting money laundering is a costly effort required by the FATF and this cost is born by the country’s reporting entities–the actors of the private sector who are in the best position to observe potential money laundering transactions and to report them appropriately [44]. Countries face the international pressure of potential blacklisting and the domestic pressure of reporting entities which want to minimize their reporting costs.

Let the payoff function for a country be

v(i)=−c*SR(i)+p(i)*S (4)

where *c* depicts the (fixed) economic costs of delivering one suspicion report to the FIU, *SR*(*i*) is the number of suspicion reports sent to the FIU of country *i*, *p*(*i*) is the probability country *i* gets blacklisted, and *S* is the sanction when being blacklisted. We assume here for simplicity that the blacklisting sanction is the same for all countries, although we are aware that [32] showed that different countries care differently about being blacklisted. Since the FATF shames and blacklists countries, the FATF’s punishment is borne by the country and not by the deviant reporting entity. Consequently, the intuition is that reporting entities put in the least effort possible while avoiding punishment. The punishment for the reporting entities can be a sanction from their supervisor for not reporting a suspicion transaction (which can be a monetary fine but can also, in some countries, include imprisonment), but can also be the reputational damage that occurs when media reports money laundering within a bank or any other reporting entity.

In essence, the situation for the obliged entity is somewhat similar to the trade-off that the countries face—more reporting is costly but reduces the reputational risk and the probability of punishment. The FATF cannot observe the true anti-money laundering effort exercised at the country level.

Instead, the FATF infers this from the number of recorded reports *SR* (see Eq 1). With minimum compliance standards (a blacklisting threshold of a certain number of suspicion reports), the FATF incentivizes through punishment. Consequently, *p*(*i*) the probability of being blacklisted is

$$p(i)=\left\{ \begin{array}{l} 0,if\;SR(i)>T\\ 1,if\;SR(i)<T \end{array}\right.$$ (5)

where *T* is the threshold number of suspicion reports as set by the FATF.

Furthermore, we distinguish two types of costs associated with reporting. [45] argues that one of the main reasons why the current anti-money laundering strategy is so unsuccessful is the general lack of understanding of money laundering by financial entities, and of how much reporting entities stand to gain from not reporting money laundering (cf. [7]). Consequently, we assume that submitting *TRs* involves high costs *c*_*H*_ and submitting *FRs* involves low costs *c*_*L*_, where *c*_*H*_ ≫ *c*_*L*_ = *c*. In thissetting, *c*_*H*_ incorporates the loss of potential high value customers and the reputation loss amongclients that value their privacy (be it for legal or illegal reasons) that the reporting entity faces when actually reporting on money laundering [46]. Conversely, *c*_*L*_ includes just the costs of filing a report–i.e. time and labor put into suspicion building, investigation, and writing of a report, such that the report meets the national standards. Consequently, the cost function of the reporting entities (in line with [6]) is

$$c*SR=-c_{H}*TR-c_{L}*FR$$ (6)

In the absence of a universal legal definition of a suspicion report, governments can affect the costs of reporting *c* = *c*_*L*_ as shown previously. SRs can be altered in terms of: the type of suspicion report; the subjective grounds for suspicion; the objective grounds for suspicion; the definition of a transaction; the inclusion of attempt; and the data collection methodology. The latter is the most vulnerable to strategic manipulation–e.g. file an SR for each suspicion transaction and person involved in a money laundering case, instead of bundling them into one SR.

### Limitations

For exposition purposes the model does not take into account all variables used by the FATF in assessing national performance in the fight against money laundering. The first blacklist was developed in 2000 based on assessing countries with regard to 25 Criteria for Identifying Countries and Territories Non-Cooperative in Anti money Laundering and Terrorist Financing [47]. We also abstract from all other geo-political motives that can underline the imposition of economic embargos–e.g. nuclear arms, political ideology, etc.

Moreover, we refrain from addressing whether it might be beneficial for a country to not fight money laundering adequately to attract illegal capital, as described in [48,49]. Finally, we also do not expose the mechanisms through which strategic alterations of national statistics are achieved–e.g. top-down pressure from the FIU to the obliged entities, horizontal peer-pressure in the professional setting, strategic cooperation between institutions etc.–at the national level.

## Results

**Fig 3:**
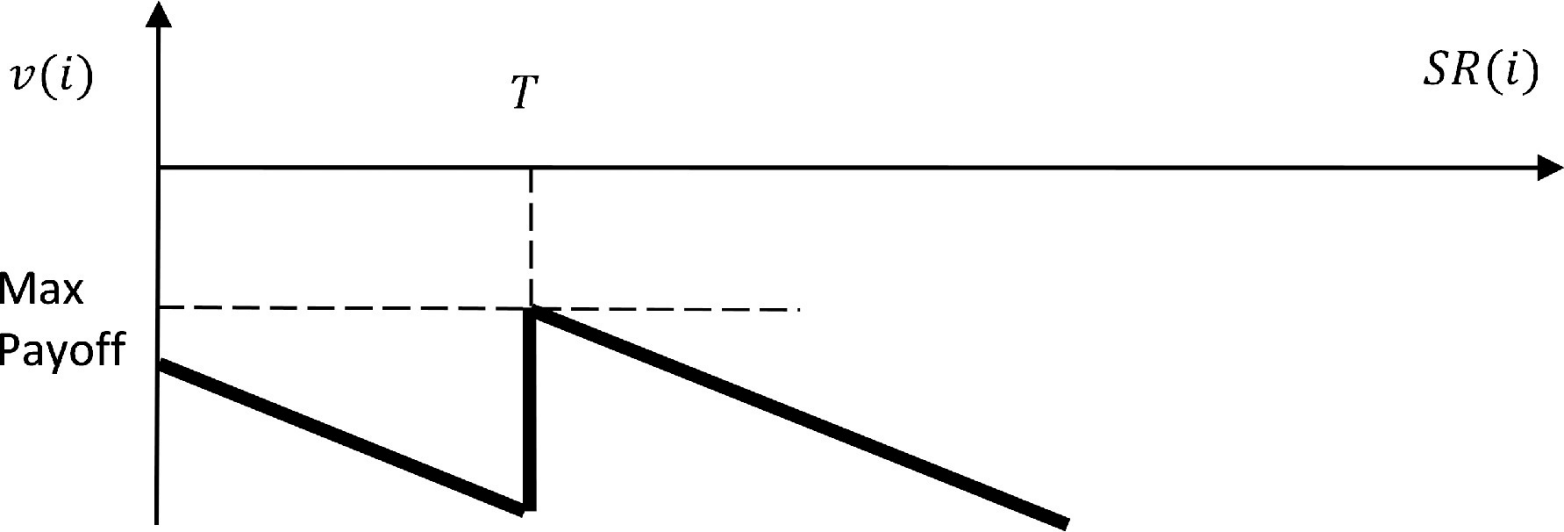
Pay-off function of the agent (country *i*). The payoff function *v*(*i*) of a country *i* is strictly negative therefore drawn in the second quadrant of the system of coordinates (payoff, number of reports). Costs increase with the number of reports *SR*(*i*) as long as the country is below the blacklisting threshold *T*. At the threshold, the costs are minimal (the payoff is maximized). Costs increase directly proportional with the number of reports, when the number of reports exceeds the minimum threshold. Since reporting entities only report false reports in this setting, the slope of the curve is equal to *c*_*L*_.

Under these assumptions, the optimization function of the government is min_*c*_ *v*(*i*). Knowing thecosts, obliged entities choose how much to report by minimizing their costs (min_*TR*,*UR*_ *c*), subject to the thresholds set by the principal. Consequently, as long as the costs of reporting false positives is lower than true positives, obliged entities will only report FRs and they will do so sufficiently much that the country is not in danger of being blacklisted. Total costs increase as more reports are filed, since every report is costly, and thus, there is no incentive to report more than the blacklisting threshold. Consequently, if the sanction of blacklisting is sufficiently high (*S* > *c* * *T*), to incentivize the agents to comply, the optimal number of suspicion reports is equal with the blacklisting threshold (see Fig 3). Finally, the payoff of the principal is

u(i)=max(TR)=0 (7)

Assuming countries are alike (in terms of money laundering threats, size of the economy through which money would be laundered, number of neighbors, costs for reporting, bureaucracy and political decision-making institutions, etc.) except for the initial number of reports *SR*(*i*), then rationalbehavior of the countries would lead to a convergence in the number of reports over time to just above the blacklisting threshold *T*. Countries with a high number of reports have an incentive to decrease the number of reports to limit the costs for the reporting entities without an additional risk of being sanctioned by the FATF. At the same time, those countries that have an insufficient number of reports should increase the number of reports to make it just above the blacklisting threshold. The same dynamics take place every time the FATF increases *T* to design a new blacklist. More SRs will be produced and the surge in reporting costs will increase domestic pressures on governments to limit reporting costs at the lowest possible level. Since this is achieved by FRs and not TRs, the number of reports converges just above the new threshold, while the payoff of the FATF remains null. Data limitations do not allow the empirical testing of this argument. Nevertheless, supporting evidence is presented in Table A in S1 Appendix.

## Implications

Insofar, the principal-agent model and the anecdotal evidence suggest that the FATF’s strategy of naming and shaming on the basis of the number of SRs is flawed. The model helps explain why the FATF blacklists are emptying in the midst of ever more impressive leaks of information on largescale money laundering and corruption schemes and can be used to propose two policy improvements: (1) unifying reporting standards and (2) using different performance indicators to compare countries.

**Fig 4:**
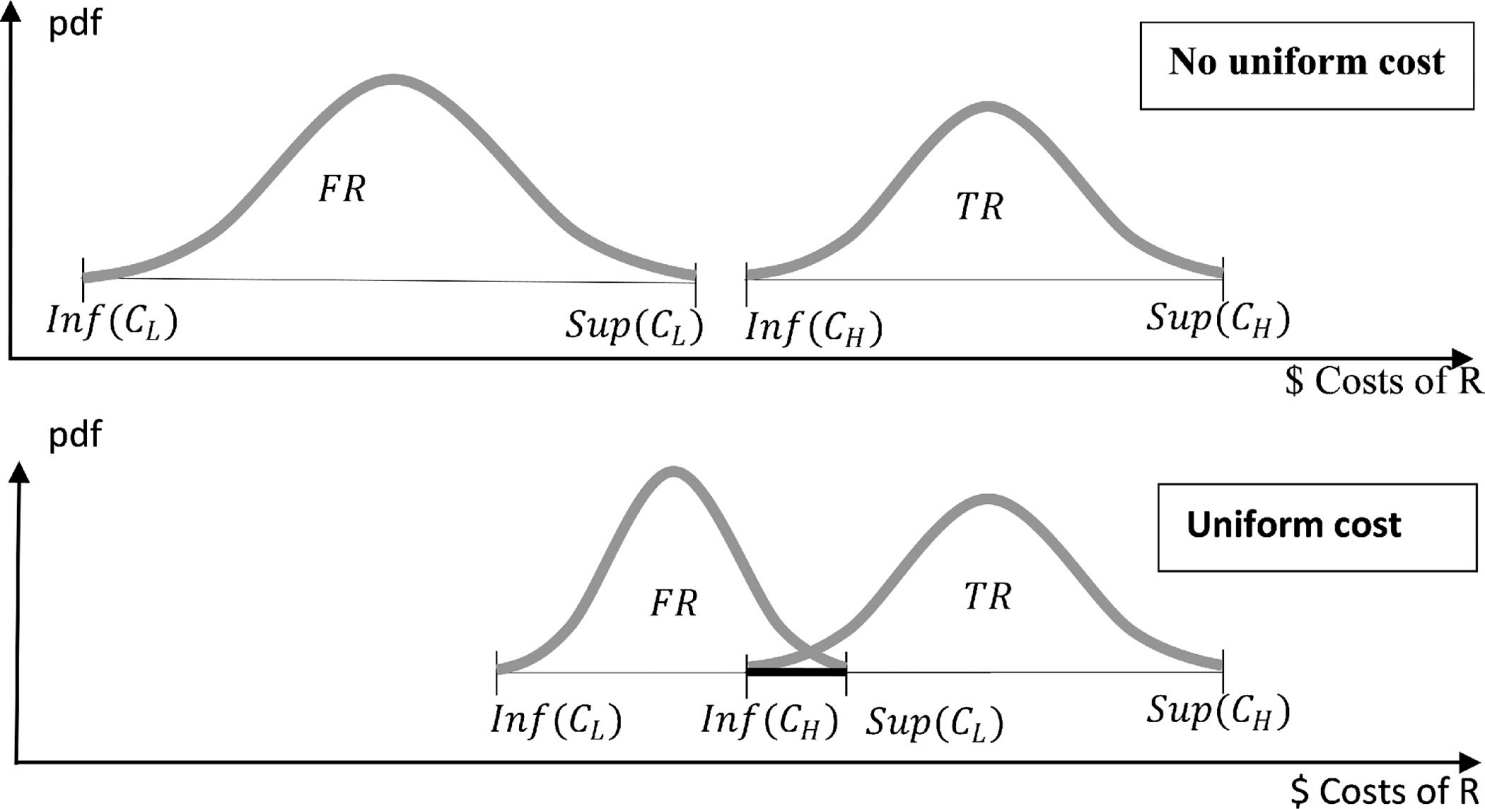
Unifying reporting standards and its impact on the cost of reporting money laundering relative to noise for reporting entities.

Harmonization requires the adoption of a single definition of an SR that covers uncertainty with respect to the client, to the transaction, that is related to a red flag or a gut feeling of the obliged entity is necessary. Similarly, harmonizing the legal definition of an SR takes away the government’s capacity to reduce reporting costs by interpreting SRs favorably and increases the effectiveness of the anti-money laundering strategy by increasing *c*_*L*_ relative to *c*_*H*_. When costs are not fixed sums but distributions within fixed intervals, unifying definitions at high standards decreases the heterogeneity of costs and increases the likelihood that reporting noise is at least as costly as reporting money launderers (see Fig 4). Consequently, when maximizing their utility function, reporting entities will optimally report some true cases of money laundering. This will, in turn, marginally raise the payoff of the FATF.

Alternatively, random tests may offer complementary evidence of the private sector’s engagement in countering money laundering. [5] conducted a randomized test with global shell companies. In a randomized control trial setting, the authors emailed more than 3700 corporate service providers globally, and asked them to set-up prohibited, untraceable or anonymous shell companies on their behalf. Their experiment revealed, among others, that setting up anonymous shell companies can be done online, fast, with ease and at low costs. These are the same ‘shell companies’ that triggered the2016 ‘Panama Papers’ scandal and that the international community explicitly forbids.

Future randomized control trials can include opening a bank account without a passport, exchanging a large sum of foreign currency, smurfing and transferring a large sum of money to a suspicious offshore company. The results of these randomized control trials can form the bedrock for FATF evaluations. While this exercise needs to be rigorously conducted and would require significant adjustments to the mandate of the FATF, we argue it is the most reliable form of evaluation. Such an exercise would create an independent indicator of effort that countries exercise in combating money laundering. Our suggestion is similar to the independent supervisor suggestion of [7] yet we believe that less biased evidence is gathered when independent researchers conduct field experiments and replicate them across countries, than when national supervisors, constrained in the national public sector hierarchy, conduct them.

Investigators would observe

$$\frac{TR}{MA}=\frac{TR}{TR+UM}$$ (8)

as described in Fig 2. In this case, the expected sanctions would be

$$S=supS*(1–\frac{TR}{TR+UM})$$ (9)

where *supS* represents the material sanction imposed oncountries that did not correctly react to any of the money laundering attempts (e.g. no suspicion report,no investigation). If countries and reporting entities are risk neutral economic agents, they wouldmaximize their utility function, which this time is min_*TR*_ *S*. This implies that all obliged entities willreport all money laundering transactions and never file a false positive, thereby maximizing the payoff of the principle *u*(*i*) = *max*(*TR*_*i*_). Our theoretical result is obviously an extreme corner solution. Consequently, we expect that conducting randomized control trials will not eliminate money laundering. Nevertheless, it has the highest potential to raise the payoff of the FATF most.

## Conclusion

In the international fight against money laundering and terrorist financing the FATF evaluates countries’ anti-money laundering policies. One of the parameters it uses is the number of reports on a suspicion of money laundering that the FIU in each country receives. The FATF uses this as a metric for the responsiveness of the private sector to the money laundering threat. The underlying assumption is that more reports are the result of the private sector truthfully reporting more. Thus, with little consideration to the risks of information pollution, countries with more reports are regarded as more effective in the fight against money laundering. The question is whether statistics like the number of suspicion reports can correctly assess the effectiveness of the fight against money laundering [12].

In this paper, we argue that modeling the country as an intermediate agent whose welfare depends on the welfare of the reporting entities allows us to solve the paradox of the ever emptying FATF blacklists. Building on the growing empirical literature that questions the reliability of national statistics due to international pressure, we argue that there is merit to not viewing national governments as proportional representations of the FATF and instead as proportional representations of their national private sector. We employ a principal-agent model with multiple agents and show that the current absence of a harmonized legal definition of a suspicion report may lead countries to engage in strategic behavior–namely to change reporting requirements such that national statistics are altered in a way that the FATF cannot punish the country for non-compliance and at the same time the reporting entities are not overburdened with costly reports.

Despite recognizing the significant differences in what constitutes a suspicion report, the FATF uses this metric of effort in their mutual evaluation reports, thereby reinforcing the rational for countries to engage in strategic behavior. On the basis of our theoretical model and analysis, we argue that the FATF has two policy options which can remedy the inefficiency of the status quo. The FATF can harmonize the definition of a suspicion report in all the countries in the world. Alternatively, the FATF can make use of randomized control trials to examine the extent to which, when faced with the same suspicious transaction, different obliged entities report their suspicion and trigger an investigation into the case.

Finally, if the FATF continues to use this metric without harmonization and without a complementary randomized control trial, we argue that more research needs to be conducted to reveal the mechanisms through which strategic behavior occurs at a national level and to reveal the magnitude thereof.

## Supporting information

S1 Appendix SR dynamics in blacklisted countries. (DOCX)
