# Supplementary material for: Strategies to avoid blacklisting: The case of statistics on money laundering
Source: PLoS One. 2019 Jun 26;14(6):e0218532. doi: 10.1371/journal.pone.0218532 (PMC6594610; doi:10.1371/journal.pone.0218532)
Supplement: S1 Appendix — (DOCX) [file pone.0218532.s001.docx]

**S1 Appendix: SR dynamics in blacklisted countries**

**Table A: Number of SRs before/ after FATF blacklisting**

| Country | Period blacklisted | SRs | 1999 | 2000 | 2001 | 2002 | 2003 | 2004 | 2005 | 2006 | 2007 | 2008 | More SRs post blacklisting? | Source |
| --- | --- | --- | --- | --- | --- | --- | --- | --- | --- | --- | --- | --- | --- | --- |
| Cook Islands | 2000-2005 | STR (total) | . | . | 4 | 3 | 19 | 14 | 15 | 29 | 29 | 30 | yes | [50] |
| Dominica | 2000-2005 | STR (total) | . | . | . | . | . | 20 | 19 | 9 | 23 | 35 | unclear | [51] |
| Philippines | 2000-2005 | STR (total) | . | . | 2 | 132 | 215 | 555 | 1048 | 4568 | 3949 | 10469 | yes | [52] |
| Egypt | 2001-2005 | STR (total) | . | . | . | . | . | 506 | 375 | 374 | 337 | 275 (half year) | no | [53] |
| Indonesia | 2001-2005 | STR (total) | . | . | 14 | 124 | 280 | 838 | 2055 | 3482 | 4454 | . | yes | [54] |
| Myanmar | 2001-2006 | STR (total) | . | . | . | . | . | 12 | 7 | 337 | 717 | . | yes | [55] |
| Myanmar | 2001-2006 | CTR (total) | . | . | . | . | . | 37 | 583 | 14425 | 42304 | . | yes | [55] |
| Grenada | 2001-2005 | SAR (total) | . | . | . | . | . | 54 | 43 | 21 | 23 | . | no | [56] |

Table A shows that obliged entities in the Philippines reported only 2 STRs just after being blacklisted, and then thousands of STRs when the Philippines were delisted and after. Similarly, in Indonesia only 14 STRs were reported at the time of the country’s blacklisting, while at the time of delisting and thereafter, obliged entities reported thousands of STRs. Finally, Cook Islands and Myanmar significantly increased the number of suspicion reports put forward by their obliged entities in the year they were delisted, and in the following years.
